# Supplementary figures and images for: Plasmodium falciparum utilizes pyrophosphate to fuel an essential proton pump in the ring stage and the transition to trophozoite stage
Source: PLoS Pathog. 2023 Dec 4;19(12):e1011818. doi: 10.1371/journal.ppat.1011818 (PMC10732439; doi:10.1371/journal.ppat.1011818)

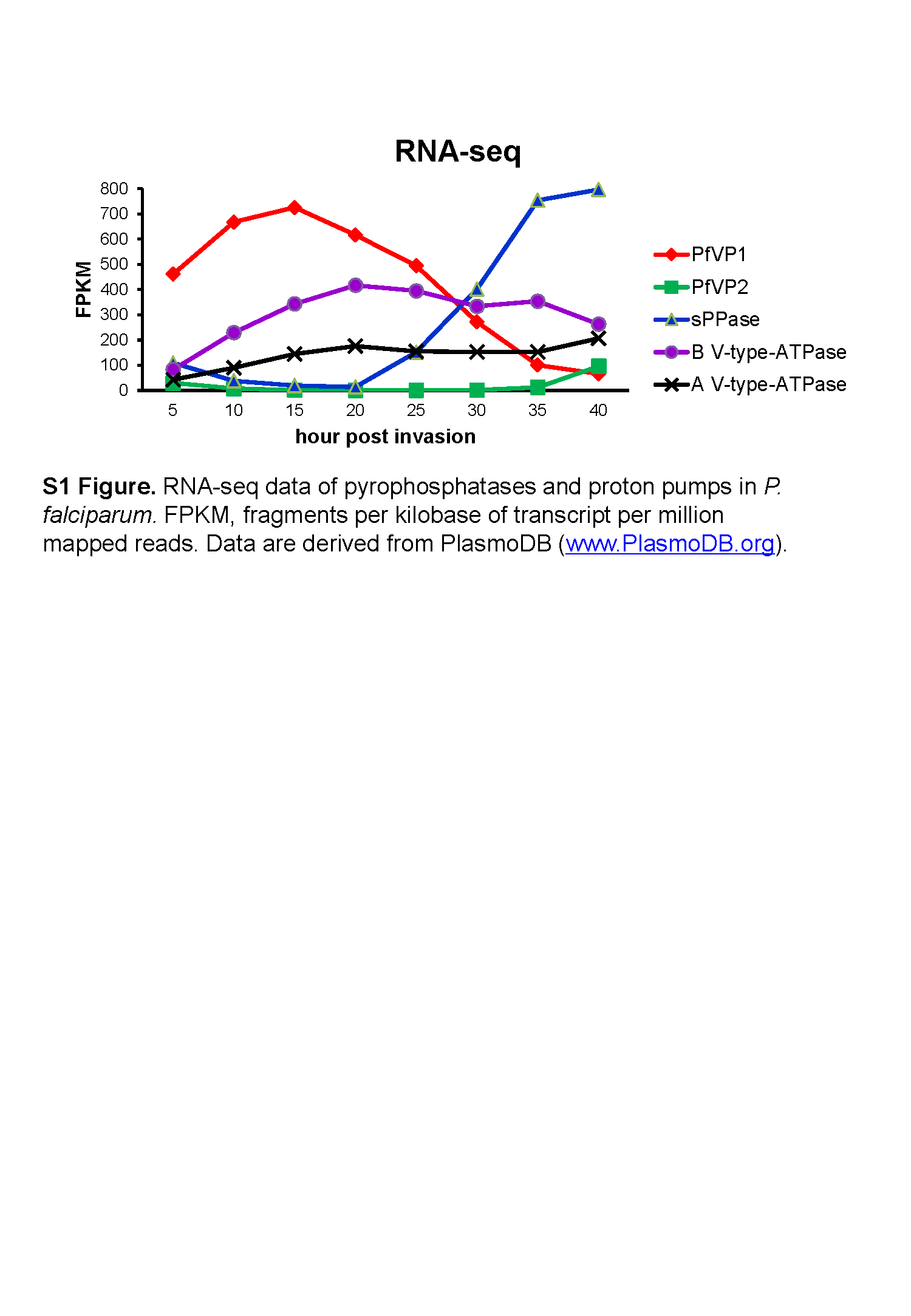

Supplement: S1 Fig — Transcription data was retrieved from PlasmoDB (deposited by Bartfai et al.[16]) and plotted. PfVP1, Plasmodium falciparum vacuolar pyrophosphatase 1; PfVP2, Plasmodium falciparum vacuolar pyrophosphatase 2; sPPase, Plasmodium falciparum soluble pyrophosphatase; V-type ATPase subunit B and subunit A. FPKM stands for fragments per kilobase of transcript per million mapped reads. (TIFF) [file ppat.1011818.s001.tiff]

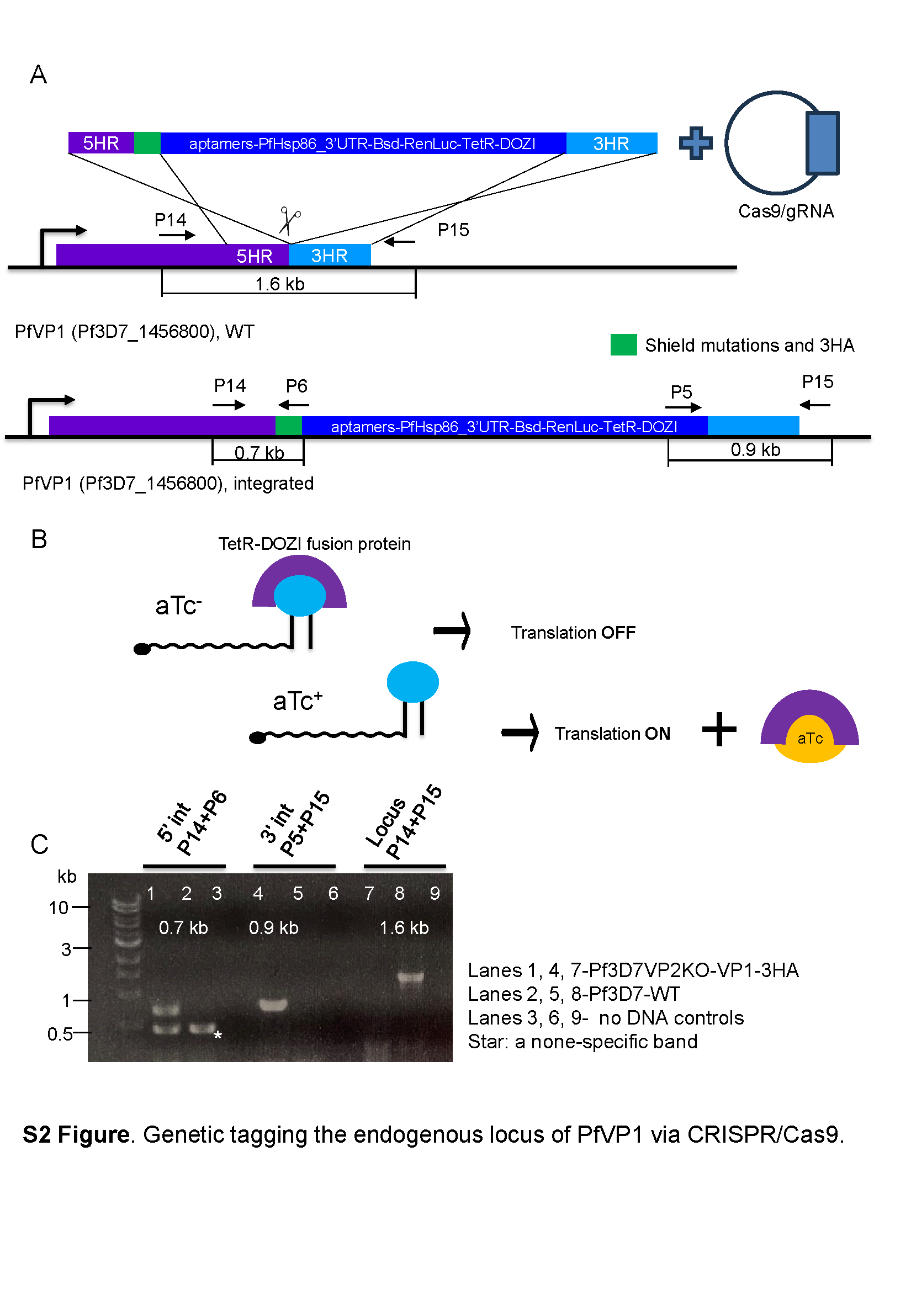

Supplement: S2 Fig — A, Model of CRISPR/Cas9 mediated gene editing. The genetic locus of PfVP1 was tagged at the C-terminal with epitopes and aptamer repeats. Blasticidin deaminase served as the transfection marker. HR, homologous region. Green box, shield mutations within the gRNA coding region and 3HA. This schematic was created with Biorender.com. B, Model of aTc regulated conditional knockdown. Without aTc, the negative regulator, TetR-DOZI fusion protein, binds to the secondary structure of aptamers and protein translation is turned off. With aTc, the transcript is freed from TetR-DOZI and translated. aTc, anhydrotetracycline. TetR-DOZI, tetracycline repressor and development of zygote inhibited. C, Genotyping of 3D7-PfVP2KO-VP1-3HAapt by PCR. 5’int, 5’ integration. 3’int, 3’ integration. Star, a non-specific band. Primer positions are shown in A. (TIF) [file ppat.1011818.s002.tif]

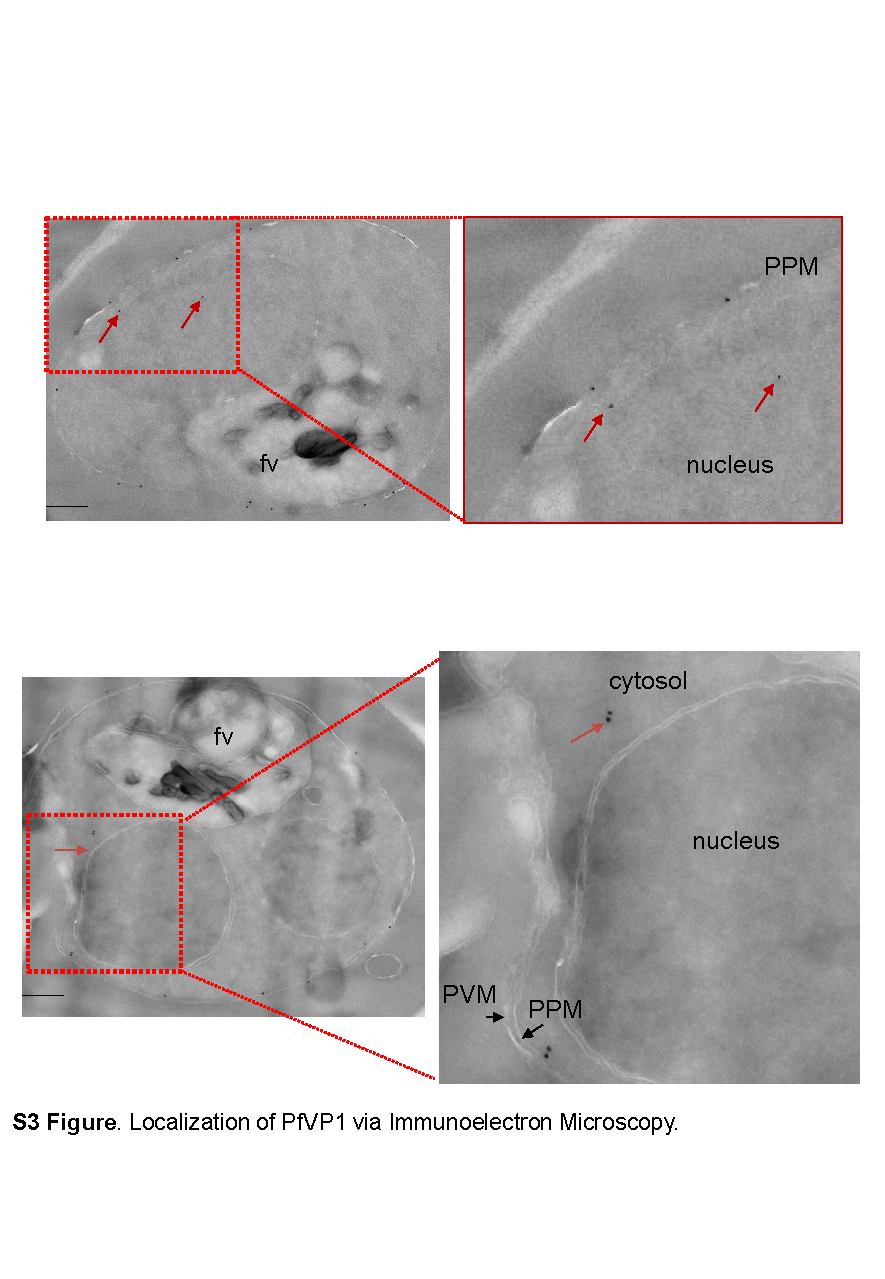

Supplement: S3 Fig — Representative immuno-EM images of 3D7-PfVP2KO-VP1-3HAapt labeled with anti-HA and gold-conjugated secondary antibodies. Black arrows indicate PVM, parasitophorous vacuolar membrane, and PPM, parasite plasma membrane. Red arrows indicate nuclear or cytosolic signals of PfVP1. Fv, food vacuole. Scale bars, 200 nm. (TIF) [file ppat.1011818.s003.tif]

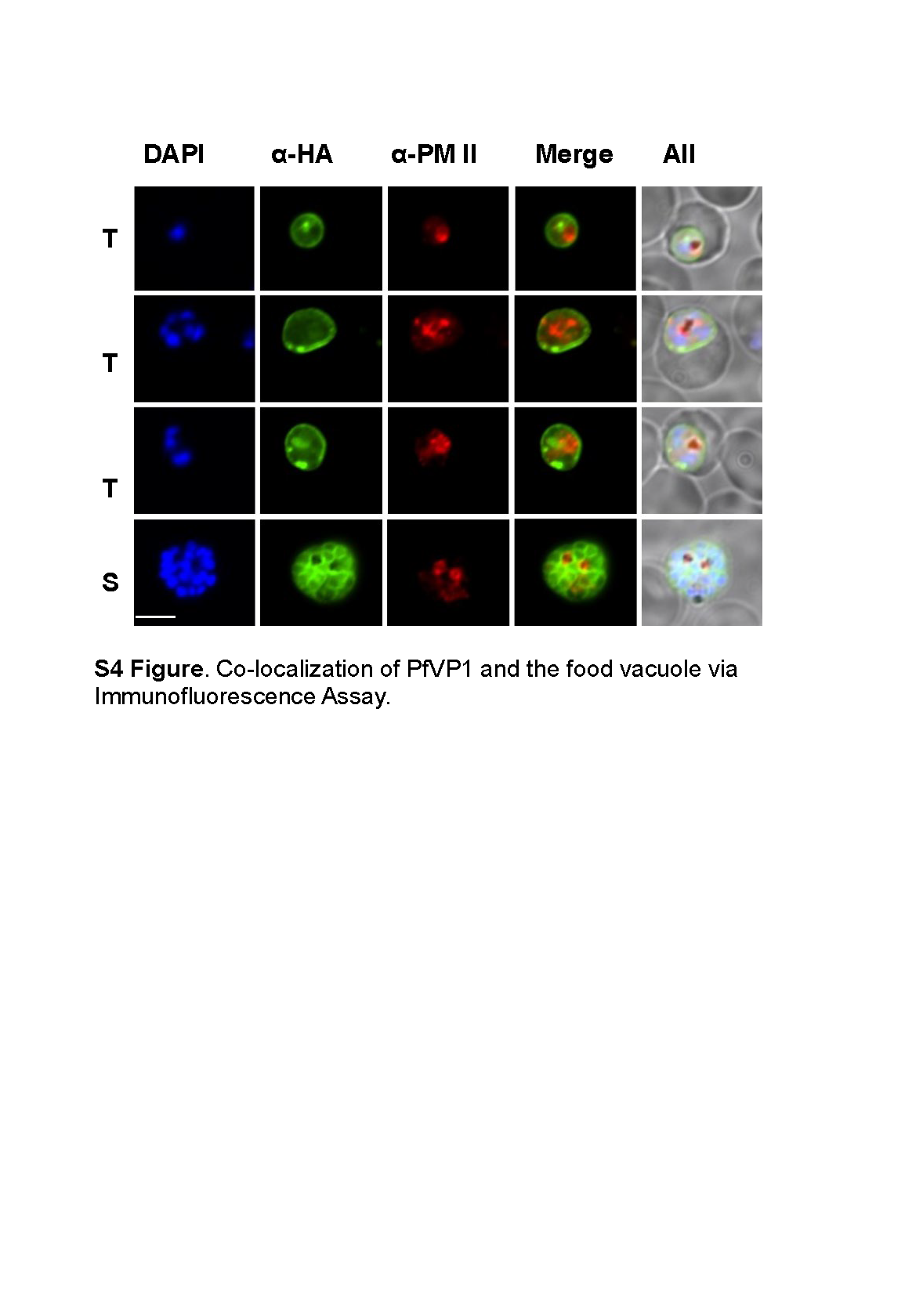

Supplement: S4 Fig — DAPI stains nuclei. PfVP1 was detected by anti-HA and Alexa Fluor 488 anti-mouse secondary antibodies. The food vacuole was detected by anti-PfPlasmepsin II [29] and Alexa Fluor 568 anti-rabbit secondary antibodies. T, trophozoite. S, schizont. Representative images of n = 25 parasites of each stage are shown here. Pearson Correlation Coefficient (0.6303±0.038) of green and red fluorescence was derived from n = 25 parasites. Scale bar, 5 μm. (TIF) [file ppat.1011818.s004.tif]

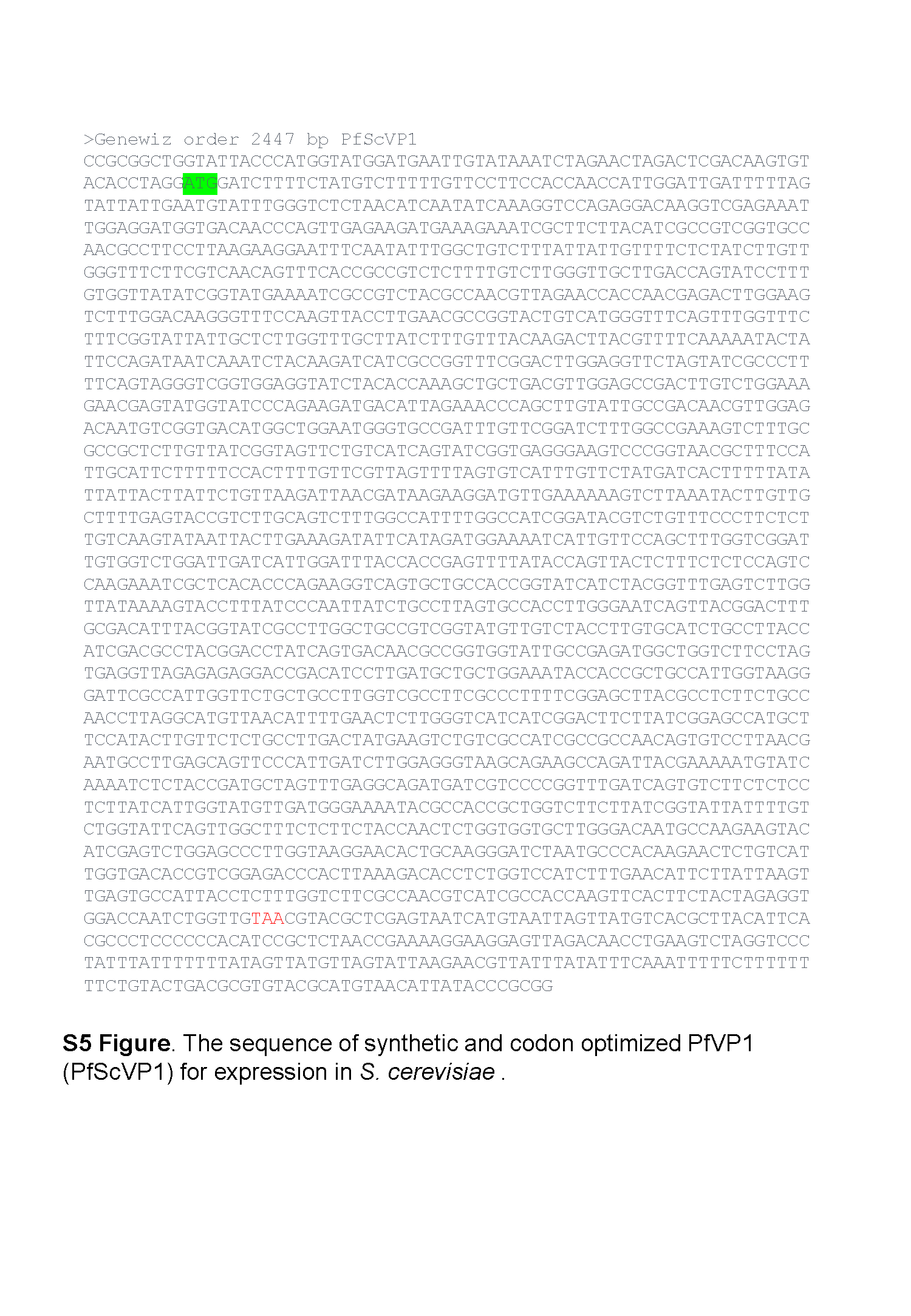

Supplement: S5 Fig — The start and stop codons of PfVP1 are highlighted in color. This synthetic DNA was made by a service company, Genewiz. (TIF) [file ppat.1011818.s005.tif]

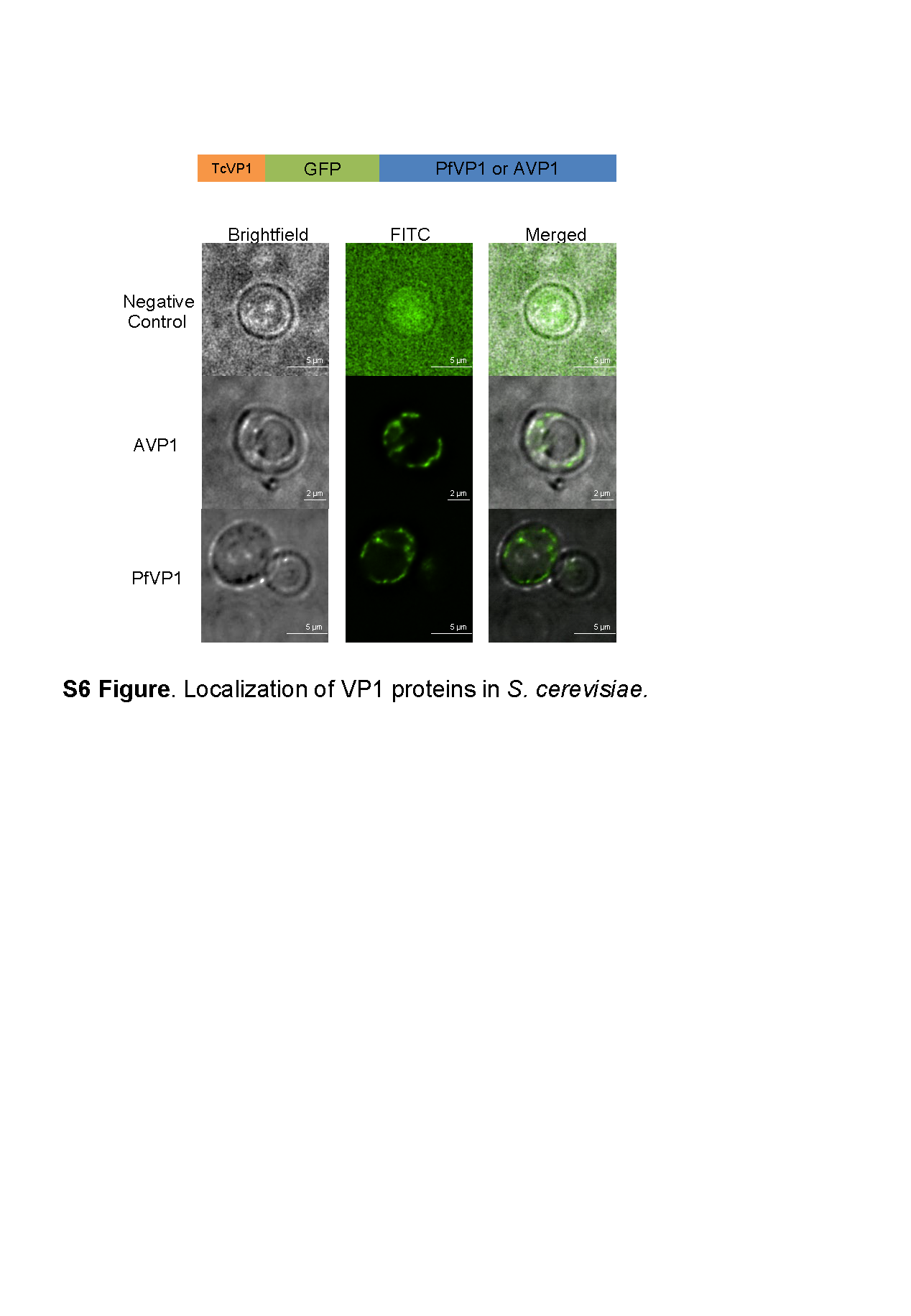

Supplement: S6 Fig — VP1 is N-terminally tagged with the localization peptide of TcVP1 (Trypanosoma cruzi) and GFP. Live cell microscopy showed localization of PfVP1 or AVP1 on the yeast’s vacuole. The yeast cell transformed with a negative control plasmid showed a fuzzy background. Representative images of n = 30 yeast cells in each condition are shown here. (TIF) [file ppat.1011818.s006.tif]

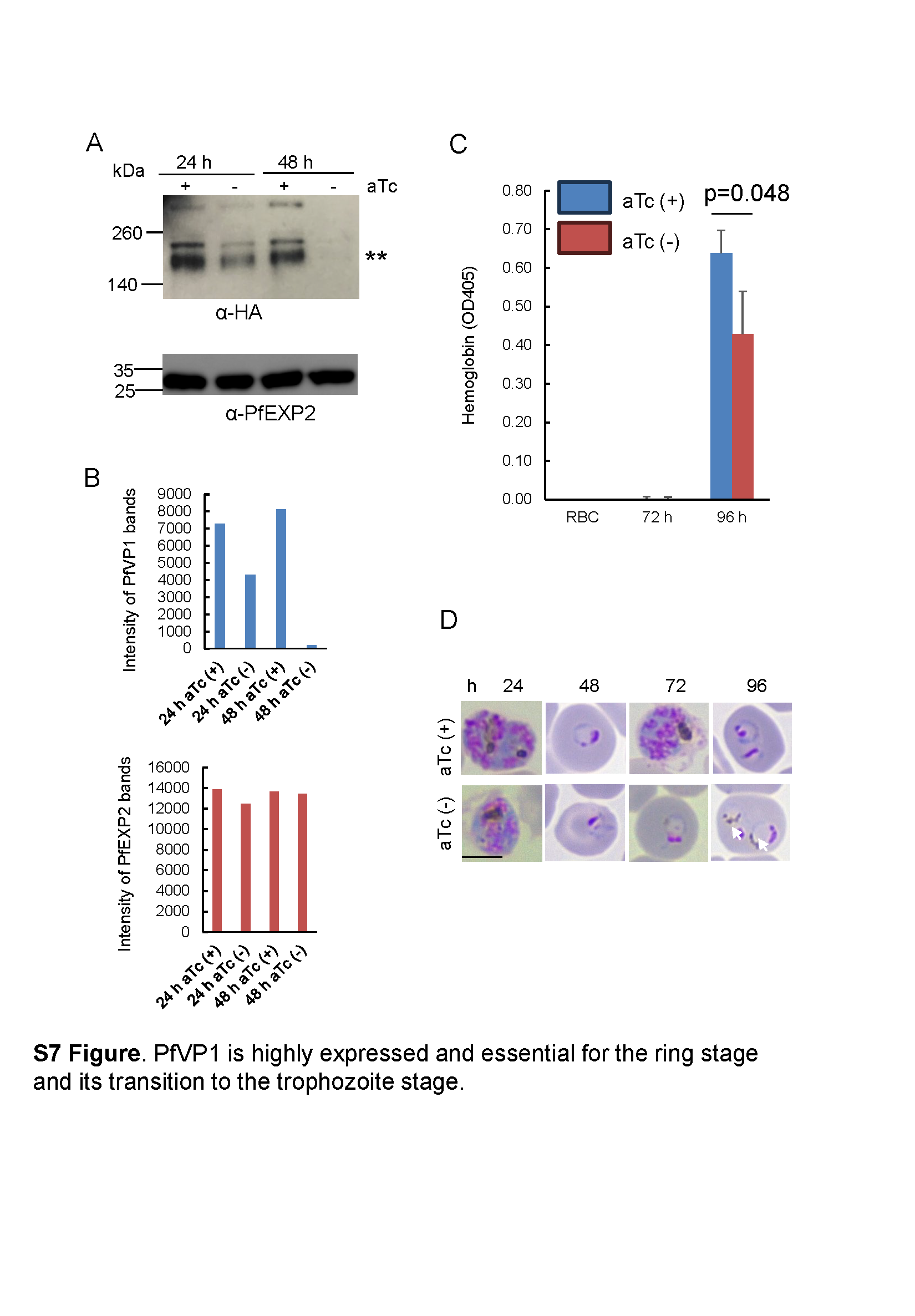

Supplement: S7 Fig — A, Western blot of 3D7-PfVP2KO-VP1-3HAapt parasites after aTc removal for 24 and 48 h from a highly synchronized culture. PfVP1 was detected by anti-HA and anti-mouse HRP conjugated secondary antibodies. PfExp2 served as a loading control. **, when a small amount of total protein lysate was loaded (~ 1 μg), only aggregated forms of PfVP1 with high molecular weights were detected by Western blot. B. The band intensity of the blots in A was quantified by ImageJ. C, The impact of alanine treatment on knockdown parasites at 72 h and 96 h post aTc removal. At each time point, parasite cultures were treated with 500 mM alanine in 10 mM HEPES and the supernatants containing hemoglobin were measured at OD405. Statistical analysis was done by Student t-test. D, Knockdown experiment starting at the ring stage by removal of aTc. Images were Giemsa-stained thin blood smears taken by a light microscope. White arrows indicate small hemozoin particles in the knockdown parasites. Scale bar, 5 μm. (TIF) [file ppat.1011818.s007.tif]

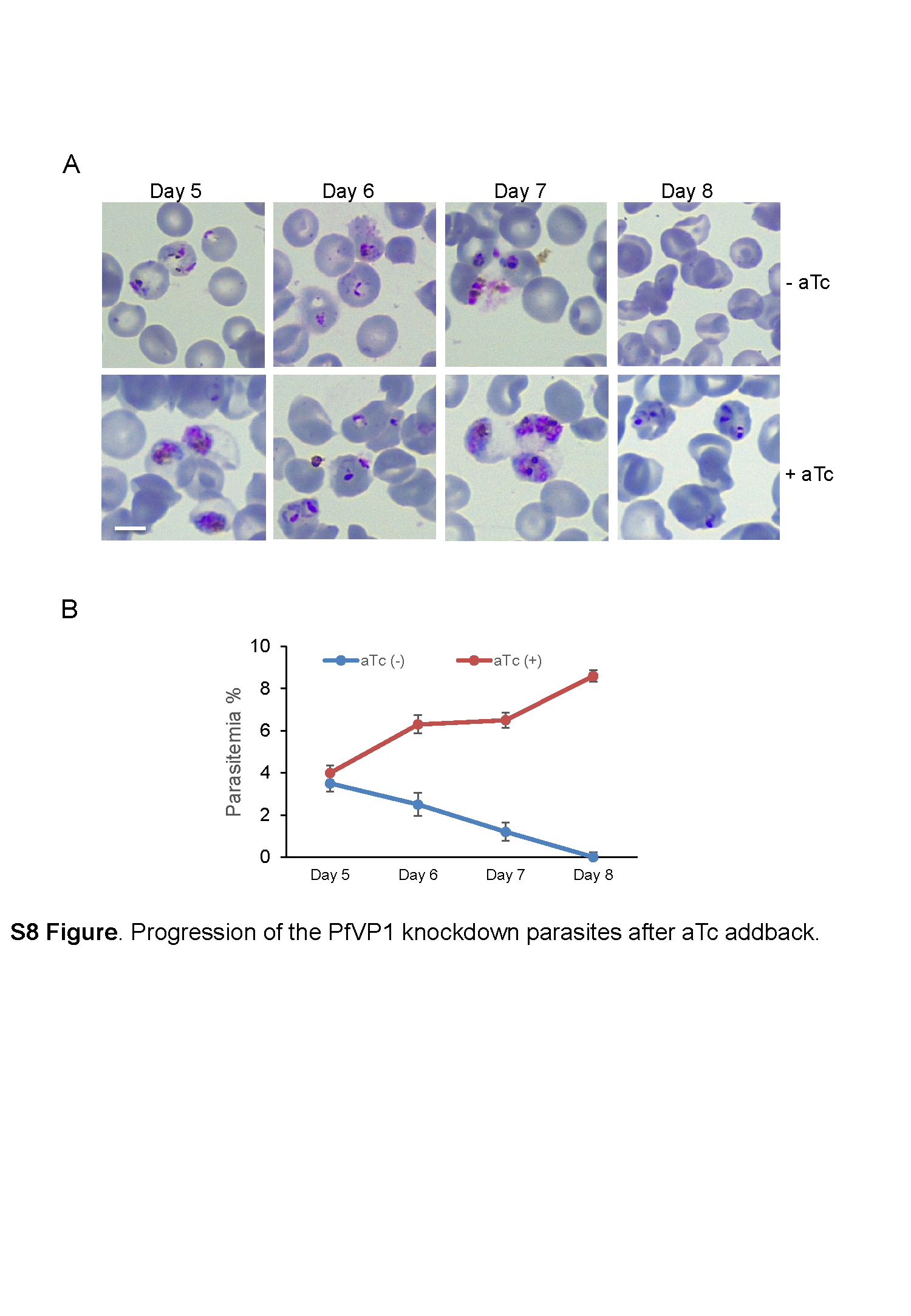

Supplement: S8 Fig — A, Giemsa-stained images show parasite morphologies upon aTc addback after it was previously removed for 96 h. Scale bars, 5 μm. B, Parasitemia of the addback cultures. Parasitemia was determined by counting Giemsa-stained thin blood smears. Mean±s.d. of three replicates are shown. This experiment was repeated four times. (TIF) [file ppat.1011818.s008.tif]

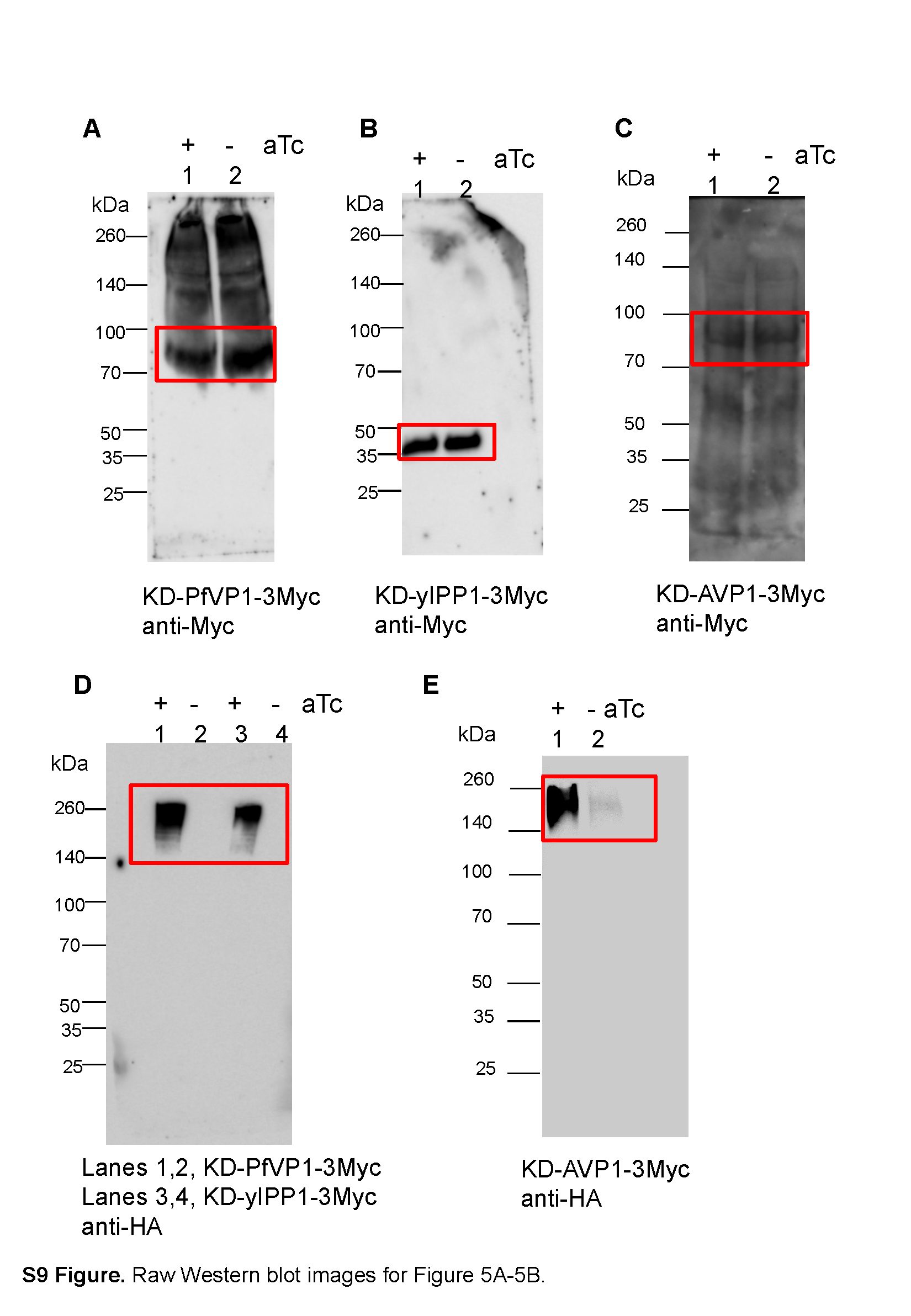

Supplement: S9 Fig — A, Expression of the episomally complemented wildtype PfVP1 protein tagged with 3Myc (A), the yIPP1 protein tagged with 3Myc (B), and the AVP1 protein tagged with 3Myc (C). In A-C, 30 μg of protein lysate was loaded in each lane. Expression of the endogenous PfVP1 protein in the wildtype PfVP1 or yIPP1 complemented parasite lines (D) and in the AVP1 complemented parasite line (E). In D-E, 3 μg of protein lysate was loaded in each lane. Lanes in red boxes were cropped and shown in Fig 5A and 5B. (TIF) [file ppat.1011818.s009.tif]

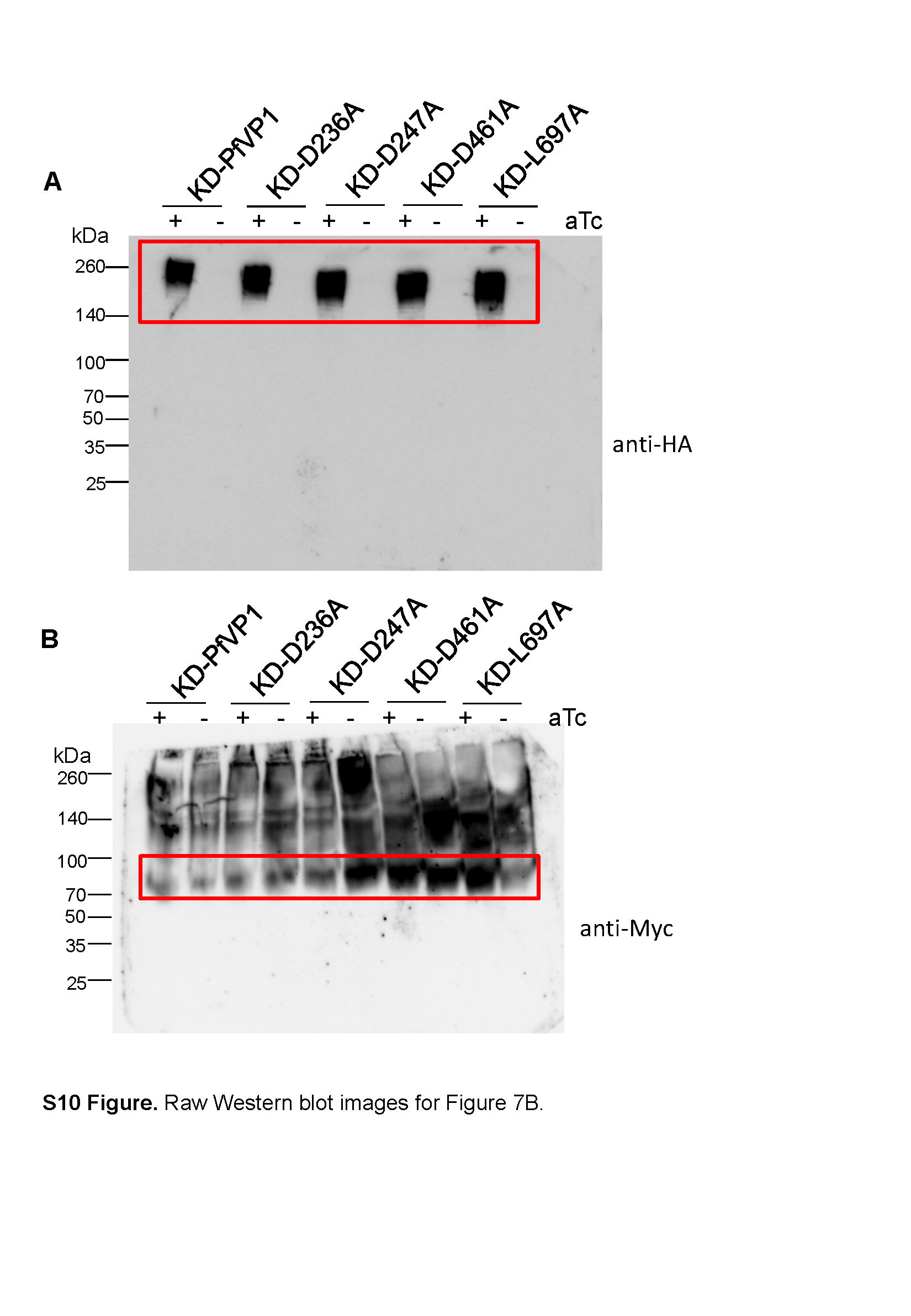

Supplement: S10 Fig — A, Western blot analysis checking the endogenous PfVP1 protein in the PfVP1 knockdown parasites complemented with wildtype or mutant PfVP1 alleles. 3 μg of protein lysate was loaded in each lane. B, Western blot analysis checking the complementary PfVP1 protein in the PfVP1 knockdown parasites complemented with wildtype or mutant PfVP1 alleles. 30 μg of protein lysate was loaded in each lane. Lanes in red boxes were cropped and shown in Fig 7B. (TIF) [file ppat.1011818.s010.tif]
