# Supplementary material for: Plasmodium falciparum utilizes pyrophosphate to fuel an essential proton pump in the ring stage and the transition to trophozoite stage
Source: PLoS Pathog. 2023 Dec 4;19(12):e1011818. doi: 10.1371/journal.ppat.1011818 (PMC10732439; doi:10.1371/journal.ppat.1011818)
Supplement: S1 Supplementary Information — (DOCX) [file ppat.1011818.s011.docx]

**Supplementary Information**

**Plasmodium falciparum utilizes pyrophosphate to fuel an essential proton pump in the ring stage and the transition to trophozoite stage**

Omobukola Solebo^1^, Liqin Ling^1,2^, Ikechukwu Nwankwo^1^, Jing Zhou^2^, Tian-Min Fu^3,4^, Hangjun Ke^1,^*

Running title: *Plasmodium falciparum* vacuolar pyrophosphatase 1

^1^Center for Molecular Parasitology, Department of Microbiology and Immunology, Drexel University College of Medicine, Philadelphia, Pennsylvania, USA

^2^Department of Laboratory Medicine, West China Hospital, Sichuan University, Chengdu, China

^3^Department of Biological Chemistry and Pharmacology, The Ohio State University College of Medicine, Columbus, Ohio, USA

^4^The Comprehensive Cancer Center, The Ohio State University, Columbus, Ohio, USA

*Correspondence: hk84@drexel.edu

**Materials and Methods**

1, Plasmid construction for *P. falciparum* studies

1) Endogenous tagging of *pfvp1* with 3HA (hemagglutinin). To modify the endogenous locus of *pfvp1* (PF3D7_1456800) via CRISPR/Cas9[1, 2], we made a template plasmid and two gRNA plasmids. Briefly, the *pfvp1* locus was tagged with 3HA and the regulatory elements (TetR-DOZI-aptamers)[3, 4] required to regulate the expression of *pfvp1*-3HA under the control of the small molecule aTc (anhydrotetracycline). For constructing the pMG75 template plasmid[5, 6], we amplified two homologous regions (5HR and 3UTR) from WT genomic DNA using primers P1-P4. These two PCR products were cleaned and annealed together by an extension PCR method[7]. The assembled 3UTR+5HR fragment was digested with BssHII and BstEII, cloned into pMG75-3HA, and sequenced using vector primers (P5-P6). These procedures resulted in the template plasmid, pMG75-PfVP1-3HA. For parasite transfection, the template plasmid was linearized with EcoRV and mixed with two circular gRNA plasmids.

For constructing gRNA plasmids, we selected the best two gRNA sequences using the Eukaryotic Pathogen CRISPR guide RNA Design Tool (http://grna.ctegd.uga.edu/) and cloned them individually into our NF-Cas9-yDHOH(-) plasmid[6], bearing Cas9 from *Streptococcus pyogenes*. Cloning of gRNAs was carried out via NEB HiFiDNA Assembly as previously described[6]. Oligo sequences are listed as P7-P10. The cloned gRNAs were sequenced using a vector primer (P11).

2) Endogenous tagging of *pfvp1* with mNeonGreen. We amplified the mNeonGreen sequence from pM2GT-Hsp101-mNeonGreen plasmid (kindly provided by Dr. Joshua Beck, Iowa State University) using P12-P13. The PCR product was digested and cloned into pMG75-PfVP1-3HA using SalI and ApaI sites. The replacement of 3HA with mNeonGreen was confirmed by sequencing using primers P12-P13.

3) Complementing PfVP1 knockdown parasites with WT or mutant PfVP1 alleles. In the D10-PfVP1-3HA^apt^ line, we performed the second transfection to add back either a WT or mutated PfVP1 allele under a strong *P. falciparum* promoter (Cam, PF3D7_1434200). To this end, we first replaced the PfmtRL2 promoter in the pLN-RL2-hDHFR-3Myc construct[5] with the Cam promoter[8], yielding pLN-Cam-hDHFR-3Myc. We then amplified WT PfVP1 from genomic DNA using P16-17 and cloned it into this plasmid via AvrII and BsiWI sites, yielding pLN-Cam-hDHFR-PfVP1-3Myc. To make individual mutant PfVP1 alleles (D236A, D247A, D461A, L697A), we introduced each mutation into primers (P18-25), amplified two homologous fragments bearing the mutation, and assembled them into a full-length fragment using NEB HiFiDNA Assembly. Each of the assembled full-length mutant PfVP1 fragments was amplified, digested, and cloned into pLN-Cam-hDHFR-3Myc via AvrII and BsiWI, resulting in 4 mutant PfVP1 plasmids. All WT and mutant PfVP1 alleles were sequenced using the pLN vector primers (P26-P27).

4) Cloning *Saccharomyces cerevisiae* inorganic pyrophosphatase (yIPP1) for complementation studies. The yeast IPP1 was gene amplified from *S. cerevisiae* genomic DNA using primers P28-29, cloned into pLN-Cam-hDHFR-3Myc via AvrII and BsiWI sites, and sequenced by primers (P26-P27).

5) Cloning *Arabidopsis thaliana* VP1 (AVP1) for complementation studies. The AVP1 gene was amplified from p426CUP1-TcGFPAVP1 (see below) using primers P30-31, cloned into pLN-Cam-hDHFR-3Myc via AvrII and BsiWI sites, and sequenced by primers (P26, P27, P32).

2, Plasmid construction for yeast studies (*Saccharomyces cerevisiae*)

1)The yeast expression vectors were kindly provided by Dr. Kendal Hirschi from Baylor College of Medicine[9]. The plasmid p426CUP1-TcGFPAVP1 bears the copper inducible promoter CUP1, which drives the expression of the N-terminally tagged AVP1. The N-terminal tag, TcGFP, is the fusion of the first 28 aa of *Trypanosoma cruzi* VP1 (TcVP1) plus the full-length green fluorescent protein (GFP), which facilitates AVP1’s localization onto yeast vesicles[10]. The plasmid p426CUP1 contains nutritional markers including his3, trp1, leu2, and ura3.

2) Cloning codon optimized PfVP1 for yeast expression. The scPfVP1 (synthetic codon optimized PfVP1 using *S. cerevisiae* codon usage tables) was synthesized by GeneWiz and cloned into a pUC plasmid (pUC-Kan-scPfVP1). The scPfVP1 sequence is also flanked by two homologous regions matching the ends of the p426CUP1-TcGFPAVP1 vector digested by BsrGI. The scPfVP1 sequence plus homologous regions was released from the pUC plasmid by SacII and cloned into p426CUP1-TcGFPAVP1 via NEB HiFiDNA Assembly. Replacement of AVP1 by scPfVP1 was verified by sequencing using primers P33-P35.

All primers used in this study were synthesized by GeneWiz and listed in **Table 1**. All cloned fragments were sequenced by Sanger sequencing (GeneWiz). All restriction enzymes were ordered from New England Biolabs.

**Table 1. Primers and oligo sequences.**

| ID | Name | Sequence (5’-3’) |
| --- | --- | --- |
| P1 | PfVP1_5HRF | GAccgcgggatatctccggaCTTTGCTTTATTTGGTGCATATG |
| P2 | PfVP1_5HRR | AAAATGTTTATCAAACCGGGggtaaccTGgtcgacCAACCAAATAGGACCACCCCTAGTAGATGTAAATTTGGTAGCAATAAC |
| P3 | PfVP1_3UTRF | ATGGCCCCTTTCCGGgcgcgcTATAGGGAACAAATCGAATATAATAAAAAG |
| P4 | PfVP1_3UTRR | tccggagatatcccgcggTCCTATGACAGTAAAAACAGTG |
| P5 | pMG75SeqF | CTTTAAATTCATGCAAAAATTTAC |
| P6 | BBHArev | GTCAGGAACGTCGTATGGATA |
| P7 | PfVP1gRNA1 | CATATTAAGTATATAATATTGCAACCAGAGGAGGACCAATAGTTTCAGAGCTATGCTGGA |
| P8 | PfVP1gRNA1N21 | GCAACCAGAGGAGGACCAATA |
| P9 | PfVP1gRNA2 | CATATTAAGTATATAATATTGTAACCATATTGGTCCTCCTCGTTTCAGAGCTATGCTGGA |
| P10 | PfVP1gRNA2N21 | GTAACCATATTGGTCCTCCTC |
| P11 | SuperCPF1_48bp | CTTTTATTTTTACTGTAATATAATTTTTTATAATGTAAAAATAAAGGG |
| P12 | NeongreenSalIF | CTgtcgacGGAAGTGGAGGAGTGAGCAA |
| P13 | NeongreenApaIR | ATgggcccTCACTTGTACAGCTCGTCCA |
| P14 | PfVP1_5fOUT | AAGGTTTTGCTATTGGTTCTG |
| P15 | PfVP1_3fOUT | GAAAAGGAGAAAACAAAATTG |
| P16 | VP1WTAvrIIF | GAcctaggATGGATCTTTTTTATGTGTTTTTATTTC |
| P17 | VP1WTBsiWIR | GAcgtacg*TCCTCCACTTCC*TAACCATATTGGTCCTCCTCTG |
| P18 | VP1D236AF | AGATAATGTAGGTGCAAATGTTGGAG |
| P19 | VP1D236AR | CTCCAACATTTGCACCTACATTATCT |
| P20 | VP1D247AF | GTATGGGTGCTGCATTATTTGGCTC |
| P21 | VP1D247AR | GAGCCAAATAATGCAGCACCCATAC |
| P22 | VP1D461AF | GCTTATGGACCAATAAGTGCAAATGCTGGTGG |
| P23 | VP1D461AR | CCACCAGCATTTGCACTTATTGGTCCATAAGC |
| P24 | VP1L697AF | CAGCAATTACATCTGCAGTTTTTGCTAATG |
| P25 | VP1L697AR | CATTAGCAAAAACTGCAGATGTAATTGCTG |
| P26 | pLNCam5’Seq | ATGTATATTTTAAACTAGAAAAGGAATAAC |
| P27 | pLN3’Seq | GTAGACCCCATTGTGAGTAC |
| P28 | yeastIPP1AvrIIF | atCCTAGGATGACCTACACTACCAGACAAATTGG |
| P29 | yeastIPP1BsiWIR | atCGTACGAACAGAACCGGAGATGAAGAACCAC |
| P30 | AVP1PLNFwdAvrII | atCCTAGGaaaaATGGTGGCGCCTGCTTTGTTAC |
| P31 | AVP1PLNRevBsiWI | taCGTACGtcctccacttccGAAGTACTTGAAAAGGATACCACC |
| P32 | AVP1_835Fwd | GCTGTCATTGCTGATAATGTC |
| P33 | P426CUP1VecF | CTGCTGCTGGTATTACCCATGG |
| P34 | P426CUP1VecR | CCTTTTCGGTTAGAGCGGATGTGG |
| P35 | ScPfVP1_715F | GGTGACATGGCTGGAATGGGTG |

**References**

1. Ghorbal M, Gorman M, Macpherson CR, Martins RM, Scherf A, Lopez-Rubio JJ. Genome editing in the human malaria parasite Plasmodium falciparum using the CRISPR-Cas9 system. Nat Biotechnol. 2014;32(8):819-21. doi: 10.1038/nbt.2925. PubMed PMID: 24880488.

2. Wagner JC, Platt RJ, Goldfless SJ, Zhang F, Niles JC. Efficient CRISPR-Cas9-mediated genome editing in Plasmodium falciparum. Nat Methods. 2014;11(9):915-8. doi: 10.1038/nmeth.3063. PubMed PMID: 25108687; PubMed Central PMCID: PMCPMC4199390.

3. Ganesan SM, Falla A, Goldfless SJ, Nasamu AS, Niles JC. Synthetic RNA-protein modules integrated with native translation mechanisms to control gene expression in malaria parasites. Nat Commun. 2016;7:10727. doi: 10.1038/ncomms10727. PubMed PMID: 26925876; PubMed Central PMCID: PMCPMC4773503.

4. Rajaram K, Liu HB, Prigge ST. Redesigned TetR-Aptamer System To Control Gene Expression in Plasmodium falciparum. mSphere. 2020;5(4). doi: 10.1128/mSphere.00457-20. PubMed PMID: 32817449; PubMed Central PMCID: PMCPMC7426165.

5. Ke H, Dass S, Morrisey JM, Mather MW, Vaidya AB. The mitochondrial ribosomal protein L13 is critical for the structural and functional integrity of the mitochondrion in Plasmodium falciparum. J Biol Chem. 2018;293(21):8128-37. Epub 20180406. doi: 10.1074/jbc.RA118.002552. PubMed PMID: 29626096; PubMed Central PMCID: PMCPMC5971461.

6. Ling L, Mulaka M, Munro J, Dass S, Mather MW, Riscoe MK, et al. Genetic ablation of the mitoribosome in the malaria parasite Plasmodium falciparum sensitizes it to antimalarials that target mitochondrial functions. J Biol Chem. 2020;295(21):7235-48. Epub 20200409. doi: 10.1074/jbc.RA120.012646. PubMed PMID: 32273345; PubMed Central PMCID: PMCPMC7247301.

7. Hilgarth RS, Lanigan TM. Optimization of overlap extension PCR for efficient transgene construction. MethodsX. 2020;7:100759. doi: 10.1016/j.mex.2019.12.001. PubMed PMID: 32021819; PubMed Central PMCID: PMCPMC6992990.

8. Nkrumah LJ, Muhle RA, Moura PA, Ghosh P, Hatfull GF, Jacobs WR, Jr., Fidock DA. Efficient site-specific integration in Plasmodium falciparum chromosomes mediated by mycobacteriophage Bxb1 integrase. Nat Methods. 2006;3(8):615-21. doi: 10.1038/nmeth904. PubMed PMID: 16862136; PubMed Central PMCID: PMCPMC2943413.

9. Scholz-Starke J, Primo C, Yang J, Kandel R, Gaxiola RA, Hirschi KD. The flip side of the Arabidopsis type I proton-pumping pyrophosphatase (AVP1): Using a transmembrane H(+) gradient to synthesize pyrophosphate. J Biol Chem. 2019;294(4):1290-9. doi: 10.1074/jbc.RA118.006315. PubMed PMID: 30510138; PubMed Central PMCID: PMCPMC6349097.

10. Perez-Castineira JR, Hernandez A, Drake R, Serrano A. A plant proton-pumping inorganic pyrophosphatase functionally complements the vacuolar ATPase transport activity and confers bafilomycin resistance in yeast. Biochem J. 2011;437(2):269-78. doi: 10.1042/BJ20110447. PubMed PMID: 21612578.
