# Supplementary material for: Plasmodium falciparum utilizes pyrophosphate to fuel an essential proton pump in the ring stage and the transition to trophozoite stage
Source: PLoS Pathog. 2023 Dec 4;19(12):e1011818. doi: 10.1371/journal.ppat.1011818 (PMC10732439; doi:10.1371/journal.ppat.1011818)
Supplement: Attachment — Submitted filename: pyrophosphate-proton pump_Ke_202307.pdf [file ppat.1011818.s012.pdf]

***Plasmodium falciparum* utilizes pyrophosphate to fuel an essential proton pump in the ring stage and the transition to trophozoite stage**

Omobukola Solebo , Liqin Ling , Ikechukwu Nwankwo , Jing Zhou , Tian-Min Fu, Hangjun Ke*

Solebo et al address the question of how the ring stage of P.f. parasite meets its energy demands and pump protons to maintain the plasma membrane potential. This is biochemically interesting because the mitochondrion is a negligible source of ATP in the blood stages and it is unclear whether glycolysis is sufficient for its energy demands. The authors hypothesized that an ATP-independent proton pump mechanism may be a major energy source during ring stages. In this study they focused on the proton pumping pyrophosphatase PfVP1 (Plasmodium falciparum vacuolar pyrophosphatase 1). Through endogenous tagging they determined its localization to the PPM and its essentiality to ring development. They used a yeast orthologous system to verify its enzymatic activity in proton pumping and PPi hydrolysis. They were able to show that PfVP1 knockdown results in a decrease in cytosolic pH and accumulation of PPi, leading to a reversible inhibition in ring to troph transition. Functional analysis through complementation of wild type and mutant enzymes further supported their hypothesis that PfVP1 works as a PPi driven proton pump. This is a well-designed study that address an interesting question through a nice combination of cellular, genetic and biochemical approaches. The writing is clear, the results convincing and include appropriate controls. I enjoy reading the manuscript and I think that it contributes significantly to our understating of parasite's metabolism especially in the ring stages. I have mostly minor comments.

1. Line 7 page 3 (Introduction but also in abstract), red blood cells should be mentioned before using RBC for the first time.
2. Line 107 - Please justify the use of 3D7-PfVP2KO (knockout) parasite line as a background for endogenous PfVP1 tagging and cKD instead of wild type parasites (as appear later).
3. Figure S2 – For clarity, mark the endogenous gene in the upper illustration (PfVP1 (Pf3D7_1456800), WT) otherwise it might seem as if the insertion is on a null mutant background. 5HR is not very clear. Also, what is the green box? Why "new aptamers". Please make the illustration more comprehensible for readers who are not well familiar with the tetR-Dozi system. Add numbers on primers in the illustration so readers can evaluate the integration test on C. Mark on A sizes in Kb that correspond to PCR fragments in C.
4. Figure 1 – How does the RNA-seq data from S1 (little to no expression in Schizont stage) correlate with the microscopy data (notable expression in schizonts)?
5. Lines 119-121 – Why would PfVP1 localize to the PVM during trophozoite stages?
6. Make sure title of S4 is the same in figure and in the legend in the WORD file.
7. In the attached Supplementary WORD file, legend for Figure S5 is missing and thus all subsequent legends are misnumbered and do not correspond to the main text and Sup figures numbering. Also, text in the legend does not match the text in the main article file, so it seems that this document needs to be edited and updated.
8. Line 161 - define AVP1.
9. S7 figure – Can't see anything in the Ponceau staining so it's hard to assess whether loading was comparable to conclude the efficiency of knockdown. Additionally, why does this blot look so differently than 1E? Where is the 70-80 kDa band that corresponds to the monomeric form? Why loading so little amount if the main form cannot be detected? Quantification of knockdown would be helpful.

10. Is the effect of IDP (imidodiphosphate) similar to PfVP1 knockdown? Does it block ring to troph transition? Couldn't it block other, unrelated pyrophosphatases or proton pumps in *P. falciparum*? Does treating parasites with IDP phenocopies PfVP1 KD like in 3A, 4B or 4C? such data (which should be quite simple to obtain) would strengthen the claim that IDP acts on PfVP1 or at least blocks a similar mechanism. Please refer to the fact that IDP in its optimal concentration leads to a similar pH reduction as PfVP1 partial knockdown (before protein levels drop). A full block of PfVP1 by IDP should lead to failure in ring development already in the first cycle.
11. Figure 7 – how come the rescue construct (Myc-tagged) migrate at 70 kDa and not 260 KDa (aggregates??) like the endogenous copy?
