# Supplementary material for: Plasmodium falciparum utilizes pyrophosphate to fuel an essential proton pump in the ring stage and the transition to trophozoite stage
Source: PLoS Pathog. 2023 Dec 4;19(12):e1011818. doi: 10.1371/journal.ppat.1011818 (PMC10732439; doi:10.1371/journal.ppat.1011818)
Supplement: Attachment — Submitted filename: Reveiew comments_point to point responses.docx [file ppat.1011818.s013.docx]

The reviewers were overall enthusiastic about the novelty and significance of this study characterizing a pyrophosphate-dependent proton pump and its essentiality for ring stage parasite development. As noted by the reviewers, the authors need to reassess the interpretations of their data and the strength of the conclusions that they draw from them. Additionally, the authors should resolve the issue raised by reviewer 2 concerning the variability of the signal ascribed to PfVP1 on Western blots in the manuscript. As suggested by the reviewer, images of full blots should be included as supplemental material.


Reviewer's Responses to Questions

**Part I - Summary**
Please use this section to discuss strengths/weaknesses of study, novelty/significance, general execution and scholarship.

Reviewer #1: Solebo et al address the question of how the ring stage of P.f. parasite meets its energy demands and pump protons to maintain the plasma membrane potential. This is biochemically interesting because the mitochondrion is a negligible source of ATP in the blood stages and it is unclear whether glycolysis is sufficient for its energy demands. The authors hypothesized that an ATP-independent proton pump mechanism may be a major energy source during ring stages. In this study they focused on the proton pumping pyrophosphatase PfVP1 (Plasmodium falciparum vacuolar pyrophosphatase 1). Through endogenous tagging they determined its localization to the PPM and its essentiality to ring development. They used a yeast orthologous system to verify its enzymatic activity in proton pumping and PPi hydrolysis. They were able to show that PfVP1 knockdown results in a decrease in cytosolic pH and accumulation of PPi, leading to a reversible inhibition in ring to troph transition. Functional analysis through complementation of wild type and mutant enzymes further supported their hypothesis that PfVP1 works as a PPi driven proton pump. This is a well-designed study that addresses an interesting question through a nice combination of cellular, genetic and biochemical approaches. The writing is clear, the results convincing and include appropriate controls. I enjoy reading the manuscript and I think that it contributes significantly to our understating of parasite’s metabolism especially in the ring stages. I have mostly minor comments.

We thank reviewer #1 for constructive and positive opinions on our manuscript. The encouraging feedback is greatly appreciated.

Reviewer #2: Solebo et al. carry out a series of carefully-conducted experiments on one of the pyrophosphate-utilising proton pumps present in the human malaria parasite P. falciparum. The authors show that the activity of this pump is essential for the ability of the parasite to transition from the ring stage to trophozoites. This is important new information for the field. I have one main issue pertaining to the experimental work (described below) and the authors have misinterpreted a relevant paper in the field. Both of these issues should be corrected before publication. In addition, some statements in the Discussion do not appear to be supported by the data presented and these should be removed or toned-down.

We thank reviewer #2 for constructive and positive opinions on our manuscript. The comments are greatly appreciated. Changes have been made according to the reviewer’s comments and advice in the revised manuscript.

Reviewer #3: Clearly written, the concise new message of this paper is that the ancient and relatively low energy compound pyrophosphate, is a source of energy for P. falciparum plasma membrane proton pump pyrophosphatase PfVP1. Approaches used included study of pyrophosphatases of different organisms such as Arabidopsis thaliana and Saccharomyces cerevisiae, and skillfully employed transfection of parasite with pyrophosphatase genes to elucidate the functions of PfVP1. The paper present new and important data about the functional role of PfVP1, the unique parasite specific drug target in the early stages of P. falciparum development.

We thank reviewer #3 for constructive and positive opinions on our manuscript.

One of the most important messages of this paper is that PfVP1, an essential protein, is critical for progression of intraerythrocytic cycle of parasite development, specifically in parasite transition from ring to trophozoite stage. Parasites depleted by PfVP1 by KD can still invade RBC, change shape inside the host cells, and digest hemoglobin but they are not able to visibly grow. One of the physiological markers of successful progression to the trophozoite stage of development is the establishing of the host cell membrane permeability (New Permeability pathway, NPP), which would be an accurate indicator of the time block inflicted by PfVP1 KO on parasite life. This information is missing, unfortunately, but data could be easily obtained in the simple sorbitol lysis assay. Certainly, the transcriptome profile would clarify the developmental time of the block in PfVP1-KD parasites, and it could give some insights into the block of parasite development, but this research may be beyond the task of the first report.

We thank the reviewer for this information. We agree with the reviewer that the blockage of parasite development can be checked with sorbitol lysis. In the revised manuscript, we performed alanine lysis that works the same as sorbitol. Both small molecules, alanine and sorbitol, enter the parasite through the New Permeability Pathway, NPP. As shown in the new supplementary Figure (S7C), the effect of alanine lysis (500 mM) can be evaluated by quantifying hemoglobin content in the culture supernatant after alanine treatment. At 72 h post knockdown, both aTc ± cultures were in the ring stage, and they did not show any effect of alanine lysis. At 96 h post knockdown, the aTc (+) culture was in the trophozoite/schizont stage and it showed good signals; the aTc (-) culture showed reduced effect of alanine lysis. This result suggested that formation of NPP upon knockdown of PfVP1 was likely impaired or delayed, but not entirely abolished. Therefore, the knockdown parasites were able to conduct some biochemical activities of an early trophozoite stage parasite. Despite this, however, their maturation was still blocked because the parasites were suffering other defects such as cytosolic pH imbalance (or acidosis) and PPi accumulation, which is expected to shut down metabolism.

We added the next text as follows.

*These results prompted us to verify whether the arrested parasites had established the New Permeability Pathway (NPP). This pathway is a result of the parasite’s ability to insert a channel known as the Plasmodium surface anion channel (PSAC) to the RBC membrane. The establishment of PSAC leads to the infected RBC releasing hemoglobin and undergoing osmotic lysis in response to high concentrations of small solutes. To investigate this, we treated the knockdown parasites at 72 h and 96 h post aTc removal with 500 mM alanine and quantified the hemoglobin content in the supernatants. As shown in S7C Fig, at 96 h post knockdown, the aTc (-) culture exhibited a lower degree of osmotic lysis than that of the control. This observation suggests that the NPP was partially established in the knockdown parasites at 96 h after aTc removal.*

However, based on the presented results, the Reviewer is not certain, that the statements like “…PPi is the critical energy source for the ring stage…”, or “the delayed ring stage” are correct, because parasites in ring stage survive the absence of PPi hydrolysis in KO parasites, and parasites could be recovered from the block. It would be reasonable to make a more cautious statement about the need for PPi and functional PfVP1 in parasite growth, which is also the beginning of parasite development cycle. In the last example it would be more accurate to say that parasites are delayed in transitioning into the trophozoite stage. If the authors agree with this argument, then the text should be corrected in multiple places, starting from the title.

We thank the reviewer for these comments. We believe our data suggests that PPi is a critical energy source for the ring stage because PfVP1 is a solely PPi dependent proton pump and PfVP1 is essential for ring stage development. Our 4 h time course experiment demonstrates a delayed ring stage development and failure of ring-to-trophozoite transition in the PfVP1 knockdown parasites. The knockdown parasites could be recovered only if aTc is given back, which again demonstrates the need of PfVP1 in the early development phase of the 48 h lifecycle. Overall, we believe our data is solid to support the main conclusion of this work.

Minor deficiencies:
1. Text could be improved in several places by more accurate wording. Line 48: death is caused by P. falciparum infection but not by all human malaria parasite infections. Line 50: the length of the developmental stages is different between parasite strains. Line 52: parasite surrounded by two additional membranes – PVM and host PM. Page 60: missing words – per one molecule of glucose… Line 70: “immature parasite” is not an accurate term. Line 71: “Recent studies” need references. Line 72: “long period post invasion” is a murky definition. Line 117: missing word in “live cell microscopy” et cetera.

We thank the reviewer for these comments. These issues have been corrected in the revised manuscript.

2. The description of PfVP1 localization is complicated with the analysis of co-localization of fluorescent signals from two proteins: PVM-embedded EXP2 and PfVP1. The close opposition of parasite and vacuolar membrane cannot be described quantitatively by fluorescent signals but requires electron microscopy analysis. The authors have, however, the sufficient fluorescent data indicating the PPM but not PVM has PfVP1: Fig 1 A shows the absence of the EXP2 in schizont PPM- invaginations decorated with PfVP1; Fig 1C clearly shows egressed merozoite with a strong VP1 fluorescence on the parasite periphery. This data is confirmed by immuno-gold EM images. Authors do not have convincing evidence of other locations of VP1, and the word “primarily localized to the PPM” is not justified in the text (line 138 and in several other locations).

We thank the reviewer for careful examination of Figure 1. In the revised manuscript, we have added more text to highlight the different localization of PfEXP2 as compared to PfVP1. For IFAs, we used EXP2 as an indicator of the parasite periphery as no antibodies targeting the PPM were available. Our data suggested PfVP1’s localization on PPM, not PVM. We further used immuno-EM and live cell microscopy to confirm PfVP1’s localization on the PPM. Overall, we believe our data on PfVP1’s localization is solid.

3. The accuracy of the statement in line 339 that “PfVP1 is the major proton pump in P. falciparum” likely needs to be rephrased. V-type ATPase is a more powerful proton pump controlling parasite pH (in trophozoites).

We agree with the reviewer that parasites use different proton pumps at different lifecycle stages. That’s why the complete sentence of Line 339 is “our data indicate that PfVP1 is the major proton pump in *Plasmodium falciparum* during ring stage development.”

**Part II – Major Issues: Key Experiments Required for Acceptance**
Please use this section to detail the key new experiments or modifications of existing experiments that should be absolutely required to validate study conclusions.

Generally, there should be no more than 3 such required experiments or major modifications for a "Major Revision" recommendation. If more than 3 experiments are necessary to validate the study conclusions, then you are encouraged to recommend "Reject".

Reviewer #1: (No Response)

We thank the reviewer very much for providing positive feedback on our manuscript.

Reviewer #2: Lines 140-149 – My main issue with the manuscript is the western blots – specifically the smearing observed, and the seemingly different sizes observed for PfVP1 in different western blots. The authors talk about a “regular amount”, but shouldn’t the amount be optimised for the protein of interest? It was not possible to see any distinct bands in the blot shown in Figure 1E. In fact, at the expected size for the protein (79 kDa) the smearing was the weakest. The authors indicate that this issue “warrants further investigation”, but I would argue that this needs to be sorted out for the present manuscript as western blots are used throughout the study with different size aggregates (depending on the amount of protein loaded and/or antibody used) being used to support their conclusions. In light of the smearing shown in the western in Figure 1E, I also think it’s inappropriate to then show highly cropped blots for most of the subsequent experiments. I suggest the authors include images of the full blots, if not in the main manuscript, then at least they should be included as supplementary information.

We thank the reviewer for these comments. The raw Western blot images for Figures 5 and 7 have been provided as supplementary Figures, S9 and S10, in the revised manuscript. The word “regular” was removed to avoid any confusion. As shown Figure 1, S9, and S10, we consistently observed that PfVP1 displayed smearing effect on the blots. This could be due to the highly hydrophobic nature of PfVP1 with 16 transmembrane helixes. However, the exact reason for this phenomenon remains unknown.

Line 134/135 – The sentence on these two lines is incorrect. The authors are encouraged to read reference 29 carefully (in particular the last paragraph of the paper), because we certainly did not say that PfVP1 is on the food vacuole. Quite the contrary, we stated that “the protein [PfVP1] was not present at detectable levels in isolated digestive vacuole preparations”. We then went on to say “This…. argues against PfVP1 being the digestive vacuole H+-PPase.” The data in the present manuscript therefore is consistent with the manuscript the authors state that their data contradicts!

We thank the reviewer for this comment. We are sorry for misunderstanding reference 29 (PMID:12427765, 2003). The authors did say PfVP1 was not detectable in isolated digestive vacuole by Western blot. Therefore, our data agrees with reference 29. To avoid any confusion, we changed the “as previously suggested” to “in agreement with the previous publication”.

Reviewer #3: see above

**Part III – Minor Issues: Editorial and Data Presentation Modifications**
Please use this section for editorial suggestions as well as relatively minor modifications of existing data that would enhance clarity.

**Reviewer #1:**

1. Line 7 page 3 (Introduction but also in abstract), red blood cells should be mentioned before using RBC for the first time.

Thanks for the comment. This has been revised accordingly.

2. Line 107 - Please justify the use of 3D7-PfVP2KO (knockout) parasite line as a background for endogenous PfVP1 tagging and cKD instead of wild type parasites (as appear later).

Thanks for the comment. We used the VP2KO line as the parental line because the PfVP2KO line allowed us to exclude any compensatory roles of PfVP2 when we knockdown PfVP1. This has been added to the revised manuscript.

3. Figure S2 – For clarity, mark the endogenous gene in the upper illustration (PfVP1 (Pf3D7_1456800), WT) otherwise it might seem as if the insertion is on a null mutant background. 5HR is not very clear. Also, what is the green box? Why “new aptamers”. Please make the illustration more comprehensible for readers who are not well familiar with the tetR-Dozi system. Add numbers on primers in the illustration so readers can evaluate the integration test on C. Mark on A sizes in Kb that correspond to PCR fragments in C.

Thanks for these comments. We are sorry for the confusion. Figure S2 has been modified according to the reviewer’s comments and the legend was also updated. New aptamers are the improved aptamers with better stability than the first-generation aptamers (Dr. Sean Prigge, PMID: 32817449). “new” has been deleted to avoid confusion since we cited the papers of both the first and second generations of aptamers in the manuscript. Primers have been named to match the primer IDs in the supplementary information. Primer positions and amplicon sizes have been marked in A.

4. Figure 1 – How does the RNA-seq data from S1 (little to no expression in Schizont stage) correlate with the microscopy data (notable expression in schizonts)?

This is a good point. The available RNA-seq data in PlasmoDB suggests that PfVP1 is transcribed at a low level in the schizont stage. However, we still detected good signals with microscopy. As protein translation involves multiple mechanisms, a stable VP1 expression could be attributed to regulators at the post-transcriptional level.

5. Lines 119-121 – Why would PfVP1 localize to the PVM during trophozoite stages?

Thanks for this comment. We are sorry for the confusion. We did not mean PfVP1 localizes to PVM. Instead, we meant to say PfVP1 appeared to colocalize with PfEXP2, the marker of PVM. This was because the close proximity of PVM and PPM is beyond the detection limit of our fluorescence microscope. That’s why we also performed Immuno-EM studies.

6. Make sure title of S4 is the same in figure and in the legend in the WORD file.

Thanks for this comment. We are sorry for the mistake. The title of S4 in the supplementary word file has been corrected.

7. In the attached Supplementary WORD file, legend for Figure S5 is missing and thus all subsequent legends are misnumbered and do not correspond to the main text and Sup figures numbering. Also, text in the legend does not match the text in the main article file, so it seems that this document needs to be edited and updated.

Thanks for this comment. We are sorry for these mistakes. All mismatches have been corrected in the main text file, supplementary text file, and figures.

8. Line 161 - define AVP1.

Thanks for this comment. This has been updated.

9. S7 figure – Can’t see anything in the Ponceau staining so it’s hard to assess whether loading was comparable to conclude the efficiency of knockdown. Additionally, why does this blot look so differently than 1E? Where is the 70-80 kDa band that corresponds to the monomeric form? Why loading so little amount if the main form cannot be detected? Quantification of knockdown would be helpful.

We thank the reviewer for these comments. To avoid any confusion, the Ponceau staining images were removed. As suggested by the reviewer, we quantified the band intensity of the blots by imageJ. The quantification data showed a ~ 50% knockdown after 24 h and ~ 98% knockdown after 48 h. So far, we have observed two unusual features of PfVP1 on Western blot. One was the smearing effect, as shown in Fig 1E. The other was the presence of the large-molecular-weigh bands, as shown in Fig 5B, Fig 7B, Fig S7. When a smaller amount of protein was loaded in Fig 5B, Fig 7B, Fig S7, only the large-molecular-weight bands were detectable. These phenomena were puzzling. We speculate the hydrophobicity of PfVP1 is the reason.

10. Is the effect of IDP (imidodiphosphate) similar to PfVP1 knockdown? Does it block ring to troph transition? Couldn’t it block other, unrelated pyrophosphatases or proton pumps in P. falciparum? Does treating parasites with IDP phenocopies PfVP1 KD like in 3A, 4B or 4C? such data (which should be quite simple to obtain) would strengthen the claim that IDP acts on PfVP1 or at least blocks a similar mechanism. Please refer to the fact that IDP in its optimal concentration leads to a similar pH reduction as PfVP1 partial knockdown (before protein levels drop). A full block of PfVP1 by IDP should lead to failure in ring development already in the first cycle.

We thank the reviewer for this comment. In our pH assays with saponin lysed trophozoites, IDP (0.2mM) caused a minor drop of cytosolic pH from ~7.3 to 7.1. This result was consistent with a previous work that used IDP to study the rodent malaria parasite, Plasmodium berghei (PMID, 10727425). It should be noted that a small drop of pH may not cause detectable defects in parasite development and growth under the standard culture condition which has lots of nutrients.

As suggested by the reviewer, we performed SYBR green assays to understand the EC50 of IDP in parasite culture. We wanted to treat the parasites with 10x-100x EC50 to understand whether IDP has the same effect on parasites as PfVP1 knockdown. However, SYBR assay revealed that IDP had little antimalarial effects (see below). IDP is soluble in water with a saturation concentration at 50 mg/ml (~ 200 mM). We managed to make an IDP stock at 500 mM. However, even starting from 500 mM, SYBR green assay revealed that parasites had nearly 100% growth at a concentration of 2 mM (the highest concentration in the assay). Therefore, we were unable to determine the EC50 of IDP in parasite culture, mainly because IDP is not a potent antimalarial compound at all. The reason for this could be due to the highly negative charge of IDP which makes it not permeable to the red blood cell membrane. This issue is likely alleviated in the pH assays as the red blood cell has been treated with saponin to punch holes in the membrane. Overall, IDP is not a good antimalarial compound. Its effects on parasite growth and development in vivo remains unclear.

Figure. SYBR green assay measuring the effect of IDP (left) or artemisinin (right, control) on parasite growth over 72 h.

11. Figure 7 – how come the rescue construct (Myc-tagged) migrate at 70 kDa and not 260 KDa (aggregates??) like the endogenous copy?

Thanks for this comment. The myc-tagged copies behaved the same as the endogenously tagged copy with HA. The full blots have been added as supplementary Figure S10.

**Reviewer #2:**

Line 46-47 – The authors should ensure that they are using the most up to date numbers here.

Thanks for this comment. This has been updated.

Line 79 – Reference required for membrane potential.

Thanks for this comment. However, no studies in literature have characterized how plasma membrane potential is maintained in the ring stage of Plasmodium. We modified the sentence to indicate that the ring stage parasite needs to maintain plasma membrane potential and cytosolic pH.

Line 80 – Reference required for static pH level.
Thanks for this comment. To avoid any confusion, the sentence has been changed from “keep cytosolic pH at a static level” to cytosolic pH.

Line 97 – Is reference 21 correct here?

Thanks for this comment. Yes, reference 21 contains the PfVP2 knockout data.

Lines 94/94 – The authors state as a fact the potassium and calcium dependence of PfVP1 and PfVP2. The authors need to clarify the basis for this statement and indicate whether or not this has been demonstrated experimentally.

Thanks for this comment. We removed this statement as it has not been well characterized in Plasmodium. We added the statement that AVP1 is potassium sensitive and AVP2 is calcium sensitive. The reference was also included.

Line 119 – The statement “Throughout the 48 h IDC, in the trophozoite stage,” needs to be fixed. The parasites are not in the trophozoite stage throughout the 48 h IDC.

Thanks for this comment. To avoid confusion, “Throughout the 48 h IDC” has been removed.

Line 136 – The sentence that starts on this line is clumsy and should be amended.

Thanks for this comment. This has been revised as follows. “In every stage of the IDC, including the merozoite, ring, trophozoite and schizont, we detected strong fluorescence of PfVP1-mNeonGreen at the periphery of the parasite. Since merozoites of free of PVM, the peripheral distribution of PfVP1 is consistent with its localization on the PPM.”

Line 138 – Not sure of the utility of Figure 1D. It actually gives the impression of cytosolic localization. I suggest the authors delete this figure.

Thanks for this comment. We checked our images and tried to reduce the background using the Nickon software. A better image was presented in Fig 1D. The cytosolic background could be because not all infected RBCs had the same 3D configurations. When a single image was taken, fluorescence background arose from slightly not focused planes.

Line 200 – “are shown”

Thanks for this comment. This has been corrected.

Line 208 – I do not believe that “meanwhile” is being used correctly here. Perhaps it should be “on the other hand”?

Thanks for this comment. “Meanwhile” has been changed to “on the other hand”.

Line 214 – “In consistent” needs to be fixed.

Thanks for this comment. This grammar issue has been corrected. Changed to “in agreement with”.

Line 216 and elsewhere – I suggest the authors should refrain from using the term “addback” as this is jargon, presumably a term the authors used when discussing the experiments in-house.

Thanks for this comment. The word “addback” has been used in literature in many cases, including Plasmodium research (PMID: 28841718, PMID: 19680449). We would like to keep it in this manuscript as it has been used by others.

Line 244 – I suggest the authors include a comment about the membrane permeability of IDP.

Thanks for this comment. IDP is highly negatively charged, which may make it not permeable to cell membranes. We tried to perform SYBR green assays with IDP and found out that IDP had no effects on killing parasites even at 2 mM. This result is consistent with the negative charge of IDP.

Line 297-298 – I think that the statement suggesting that, based on their data, VP1 is functionally conserved throughout evolution, is far too strong and should be deleted.

Thanks for this comment. This statement has been changed to “suggesting the functional conservation between PfVP1 and AVP1”.

Line 339 – Seems very strange to start the Discussion section with “In summary”, especially because the last paragraph of the Discussion (line 415) also starts with “In summary”.

Thanks for this comment. “In summary” has been removed.

Line 339 – On what basis do the authors conclude that PfVP1 is “the major proton pump”? I’m not sure such a conclusion can be made. Yes, it is an important pump, but there could be another that is even more important (e.g. one that results in the death of ring-stage parasites (rather than their arrest) if it is functionally knocked out/down).
Thanks for this comment. “Major” has been changed to “critical”.

Line 383 – Should be reference 53.
Thanks for this comment. The reference has been corrected.

Line 384 – I don’t think that reference 54 (a review) is appropriate reference here.

Thanks for this comment. In this review paper (#54), Hagai Ginsburg estimated the proton pump rate of V-ATPase compared to V-PPase. Please check below.

*“The capacity of the V-ATPase can be estimated [6] to be ~2.9 mM min^−1^ at 37°C (i.e. below the rate of proton extrusion by the lactate:H+ symporter). The rate of the major V-PPase is ~5 µmol mg^−1^ per hour per PPi hydrolyzed [8], from which it is possible to calculate (assuming that membrane proteins constitute ~1.5% of the total 100 g protein per l of parasites) a proton pumping rate of 0.12 mM min^−1^ (the second PPi-driven pump is much less active).”*

Line 396-397 – On what basis is the conclusion regarding the membrane potential made? I don’t believe the authors have data to support this statement.

Thanks for this comment. We agree with the reviewer that direct measurement of plasma membrane potential has not been carried out in this study. We have changed the lines (396-400) as follows.

*“**We speculate that PfVP1’s contribution to ring stage development could be multifactorial, fulfilling tasks such as maintaining cytosolic pH, establishing plasma membrane potential (Δψ), and redirecting ATP towards other energy-costly processes. Future studies are required to test these hypotheses. We acknowledge the challenges posed by working with ring stage parasites. For instance, the gold standard method of measuring Δψ using patch clamp electrophysiology has limited applicability to intracellular organisms like Plasmodium. The small size of a ring stage parasite further diminishes the feasibility of employing this technique. Clearly, alternative methodologies must be explored to thoroughly characterize ring stage biology in future.”*

Line 399 – Patch clamping of an intracellular organism has been achieved, but the ring stage is probably too small for the current setups. I believe this is the main problem.

Thanks for this comment. We agree with the reviewer.

The captions for the supplementary figures included in the supplementary file become out of order part way through because the caption for S5 is missing. I believe the same captions are correctly included in the main text.

Thanks for this comment. Sorry for the mistake. This issue has been resolved.

Reviewer #3: see above

PLOS authors have the option to publish the peer review history of their article ([what does this mean?](https://journals.plos.org/plospathogens/s/editorial-and-peer-review-process#loc-peer-review-history)). If published, this will include your full peer review and any attached files.


If you choose “no”, your identity will remain anonymous but your review may still be made public.


**Do you want your identity to be public for this peer review?** For information about this choice, including consent withdrawal, please see our [Privacy Policy](https://www.plos.org/privacy-policy).

Reviewer #1: No

Reviewer #2: **Yes:** Kevin J Saliba

Reviewer #3: No

Figure Files:

While revising your submission, please upload your figure files to the Preflight Analysis and Conversion Engine (PACE) digital diagnostic tool, [https://pacev2.apexcovantage.com](https://pacev2.apexcovantage.com/). PACE helps ensure that figures meet PLOS requirements. To use PACE, you must first register as a user. Then, login and navigate to the UPLOAD tab, where you will find detailed instructions on how to use the tool. If you encounter any issues or have any questions when using PACE, please email us at [figures@plos.org](mailto:figures@plos.org).

Data Requirements:

Please note that, as a condition of publication, PLOS' data policy requires that you make available all data used to draw the conclusions outlined in your manuscript. Data must be deposited in an appropriate repository, included within the body of the manuscript, or uploaded as supporting information. This includes all numerical values that were used to generate graphs, histograms etc.. For an example see here on PLOS Biology: <http://www.plosbiology.org/article/info%3Adoi%2F10.1371%2Fjournal.pbio.1001908#s5>.

Reproducibility:

To enhance the reproducibility of your results, we recommend that you deposit your laboratory protocols in protocols.io, where a protocol can be assigned its own identifier (DOI) such that it can be cited independently in the future. Additionally, PLOS ONE offers an option to publish peer-reviewed clinical study protocols. Read more information on sharing protocols at <https://plos.org/protocols?utm_medium=editorial-email&utm_source=authorletters&utm_campaign=protocols>

*In compliance with data protection regulations, you may request that we remove your personal registration details at any time.* [*(Remove my information/details)*](https://www.editorialmanager.com/ppathogens/login.asp?a=r)*. Please contact the publication office if you have any questions.*
