# Supplementary material for: Plasmodium falciparum utilizes pyrophosphate to fuel an essential proton pump in the ring stage and the transition to trophozoite stage
Source: PLoS Pathog. 2023 Dec 4;19(12):e1011818. doi: 10.1371/journal.ppat.1011818 (PMC10732439; doi:10.1371/journal.ppat.1011818)
Supplement: Attachment — Submitted filename: Letter to reviewer comments.docx [file ppat.1011818.s014.docx]

Dear Editorial team of PloS Pathogens:

We thank the reviewers for their constructive comments and opinions. We have revised the manuscript according to the review recommendations.

During the first round of revision, all reviewers were satisfied with the revised manuscript and additional data we provided. Dr. Kevin J. Saliba was still concerned about the Western blot data. He suggested that the smearing band pattern of PfVP1 in Western blot and the different sizes of PfVP1 in different blots should be addressed. We thank Dr. Saliba for his time and comments. We provided possible explanations to address these two issues in the revised manuscript as follows.

*In the 3D7-PfVP2KO-PfVP1-3HA^apt^ line, we also revealed that PfVP1 was highly expressed by Western blot (Fig 1E). We detected strong signals of PfVP1 in the transgenic parasite lysate, which were absent in the control lysate. Despite being specific, the band pattern of PfVP1 was highly unusual, ranging from the monomeric form of approximately 79 kDa to large, aggregated forms with molecular weights close to 260 kDa. This smearing band pattern of PfVP1 indicated that PfVP1 proteins were not completely solubilized by 2% SDS treatment. While the exact reasons for this phenomenon remain unclear at present, we speculate two possible explanations. One possibility is that the extremely hydrophobic nature of PfVP1, which possesses 16 transmembrane helices, may lead to solubility issues even in the presence of SDS. The other possibility is that PfVP1 works as oligomers in the parasites. Structural studies have shown that the orthologous VP1 protein in mung beans forms a homodimer to hydrolyze the substrate and pump protons. It is plausible that PfVP1 works as dimers or other oligomeric structures in P. falciparum, which are not entirely solubilized with 2% SDS. This intriguing characteristic of PfVP1 warrants further investigation.*

*In* ***S9A Fig****, we once again observed the smeared band pattern of PfVP1 tagged with 3Myc in Western blot, which was consistent with the band pattern of PfVP1-3HA shown in* ***Fig 1E****. As mentioned earlier, we speculated that PfVP1 proteins were not well solubilized by 2% SDS, which resulted in this highly unusual migration pattern. Notably, when a smaller amount of protein was loaded (3 µg in* ***S9D-E Fig*** *compared to 15 µg in* ***Fig 1E)****, we could only detect the PfVP1 bands with larger molecular weights, but not the forms at monomeric sizes. This implied that the larger forms of PfVP1 predominated. Despite this unusual band pattern, C-terminal tagging of PfVP1 did not interfere with parasite physiology. This finding is consistent with other VP1 orthologs in Kinetoplastids and Toxoplasma gondii, which also utilized C-terminal tagging.*

*In consistent with* ***S9 Fig****, we detected larger forms of PfVP1-3HA in a small amount of total protein lysate (3 µg) and observed PfVP1-3Myc migrated in the unusual, smeared pattern.*

In addition, we added information about protein loading of all Western blot data in Figure legends, including Fig 1E, Fig 5A, Fig 5B, Fig 7B, Fig S7A, Fig S9 and Fig S10. This information indicates that we loaded various amounts of protein in different experiments, explaining why PfVP1 was detected at ‘different’ molecular weights. Our data suggests that PfVP1 proteins were not well solubilized by 2% SDS and the larger forms predominated. This phenomenon could be attributed to the highly hydrophobic nature of PfVP1, but other possibilities may exist.

Thank you for considering the possibility of publishing our manuscript. Looking forward to hearing your feedback.

Sincerely,

Hangjun Ke, PhD

Assistant Professor

Drexel University

Department of Microbiology and Immunology

2900 Queen Lane, Philadelphia, PA
